# Supplementary figures and images for: Colored Motifs Reveal Computational Building Blocks in the C. elegans Brain
Source: PLoS One. 2011 Mar 7;6(3):e17013. doi: 10.1371/journal.pone.0017013 (PMC3049772; doi:10.1371/journal.pone.0017013)

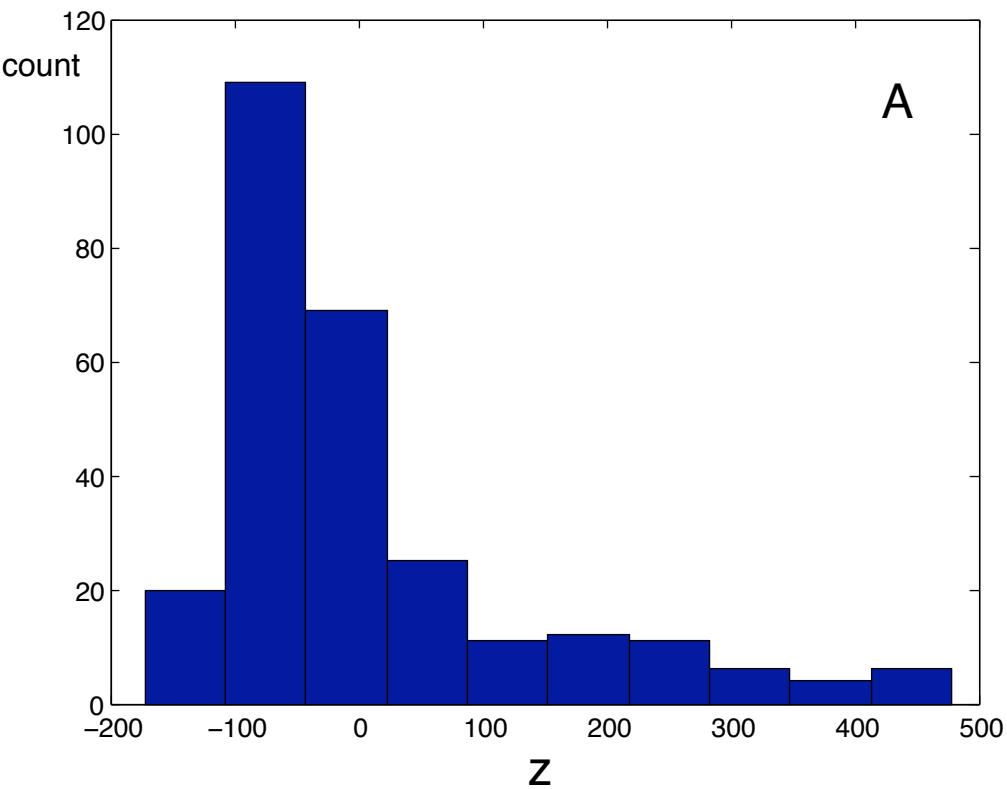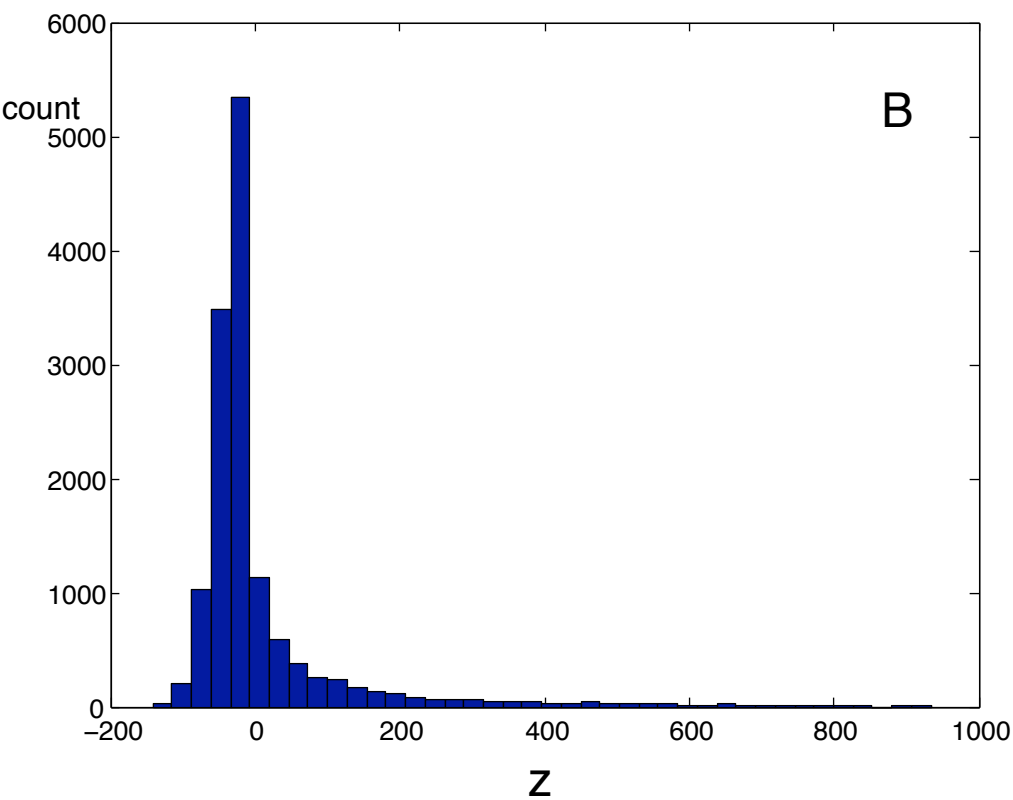

Supplement: Figure S1 — Histogram of normalized -scores of colored motif counts, where is the standard deviation of the count distribution with randomizations. A: motifs of size 3, B: motifs of size 4. (PDF) [file pone.0017013.s001.pdf]

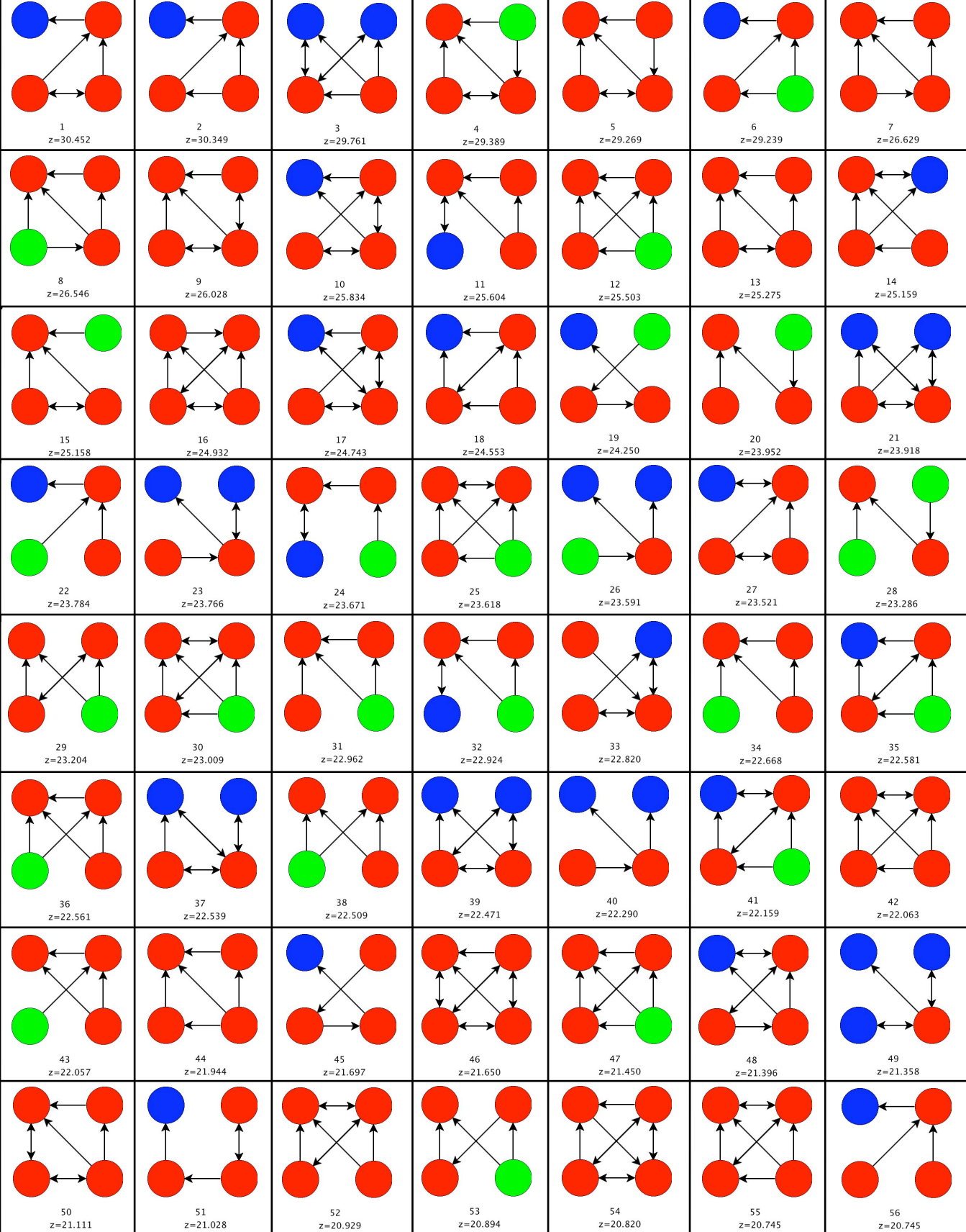

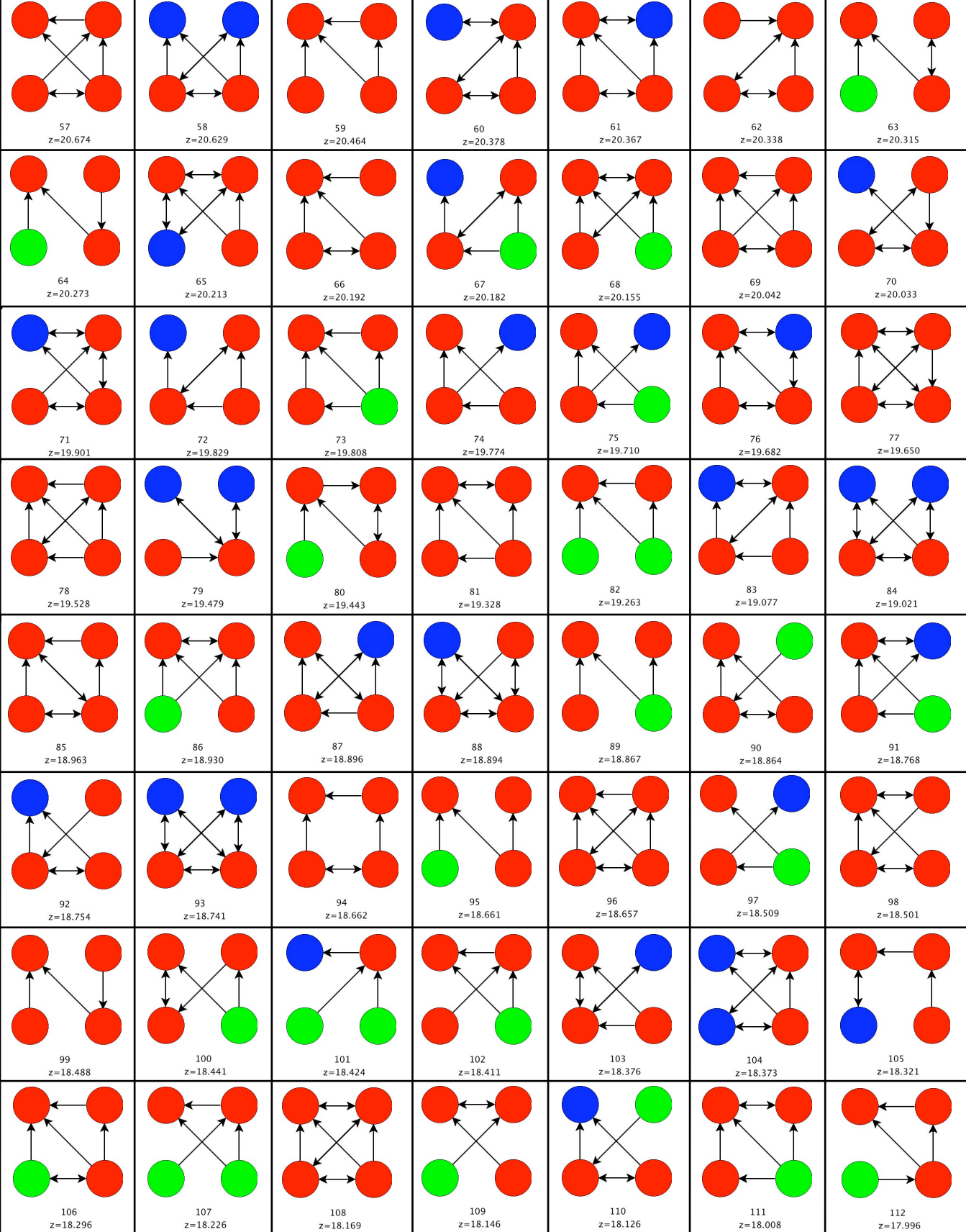

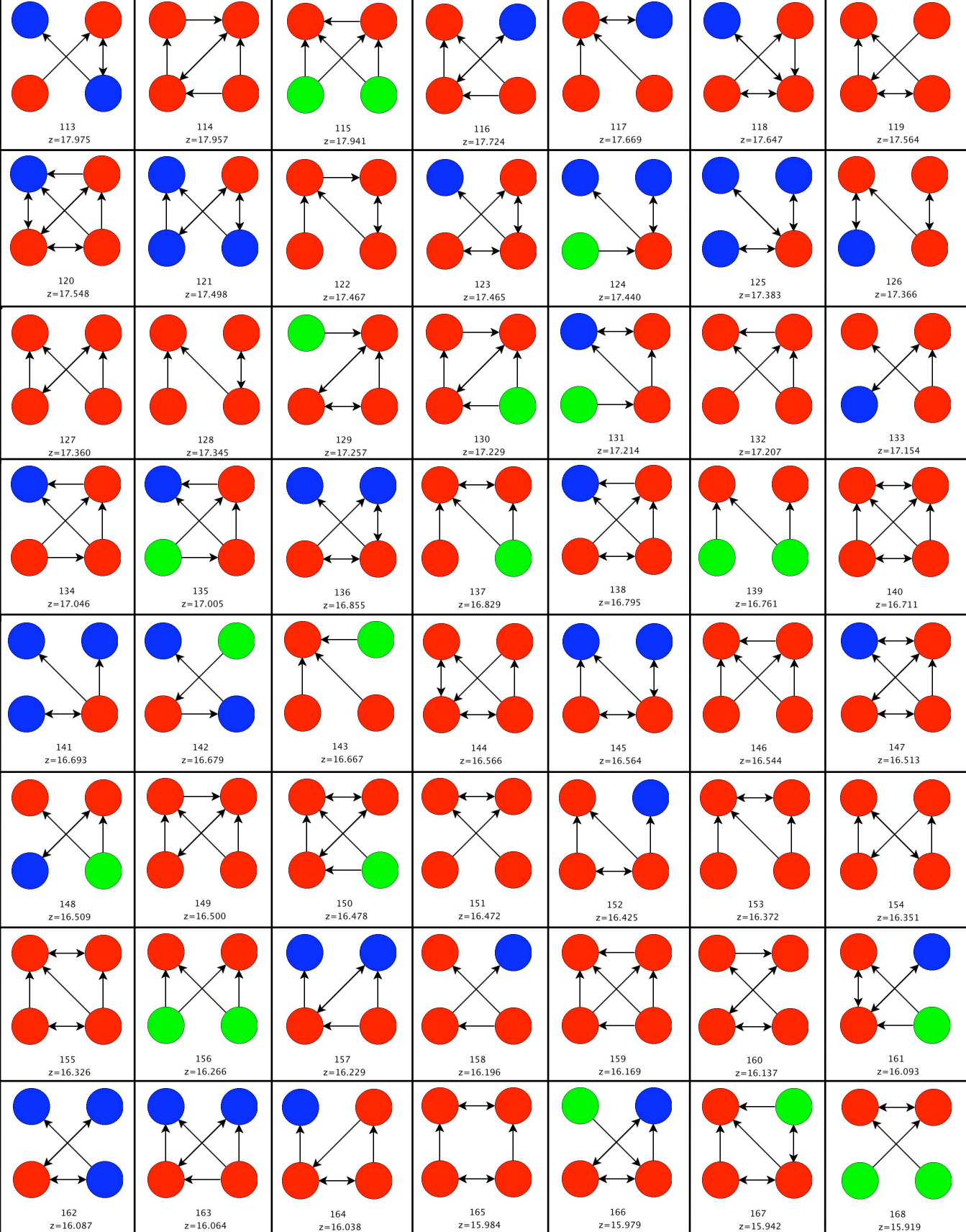

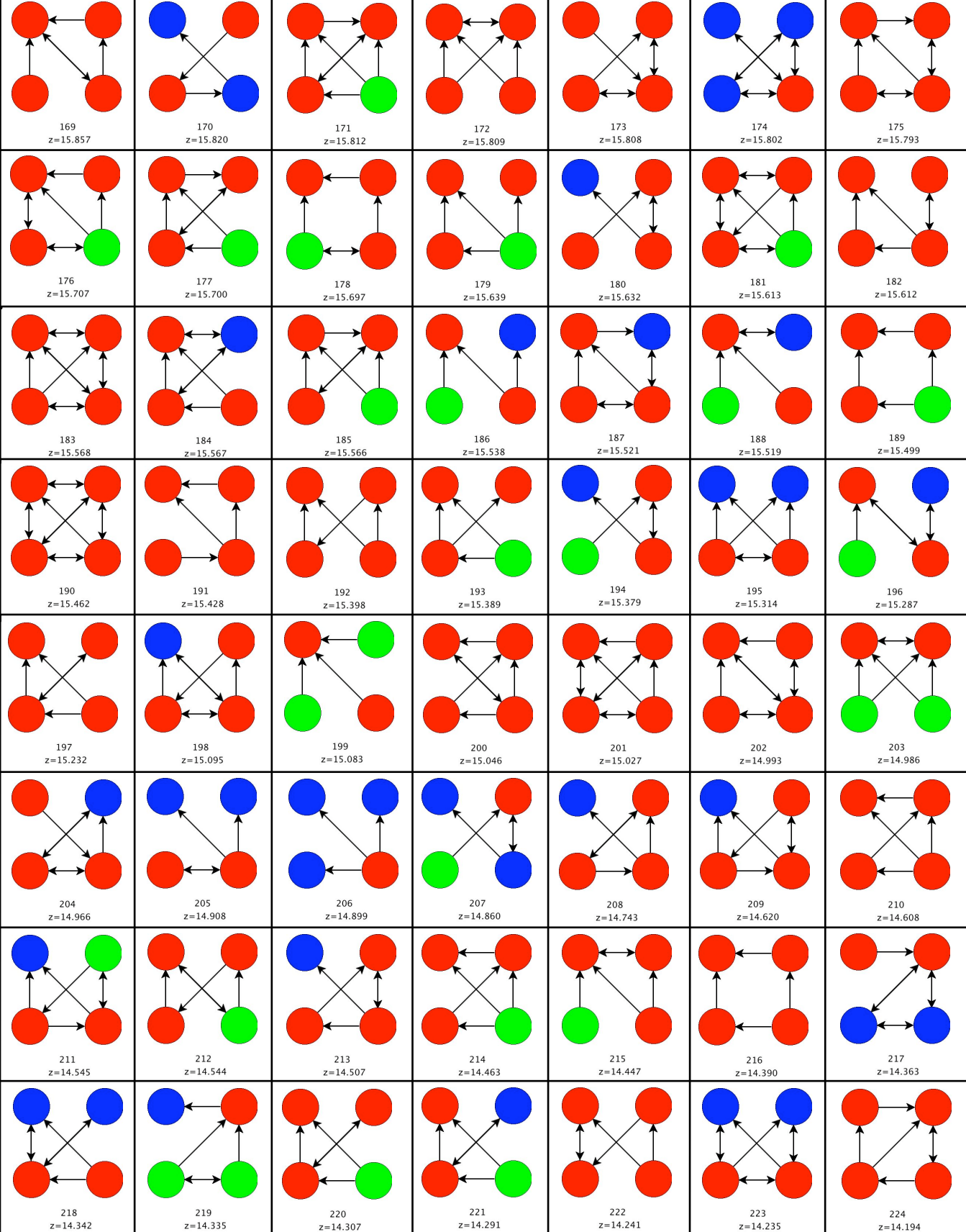

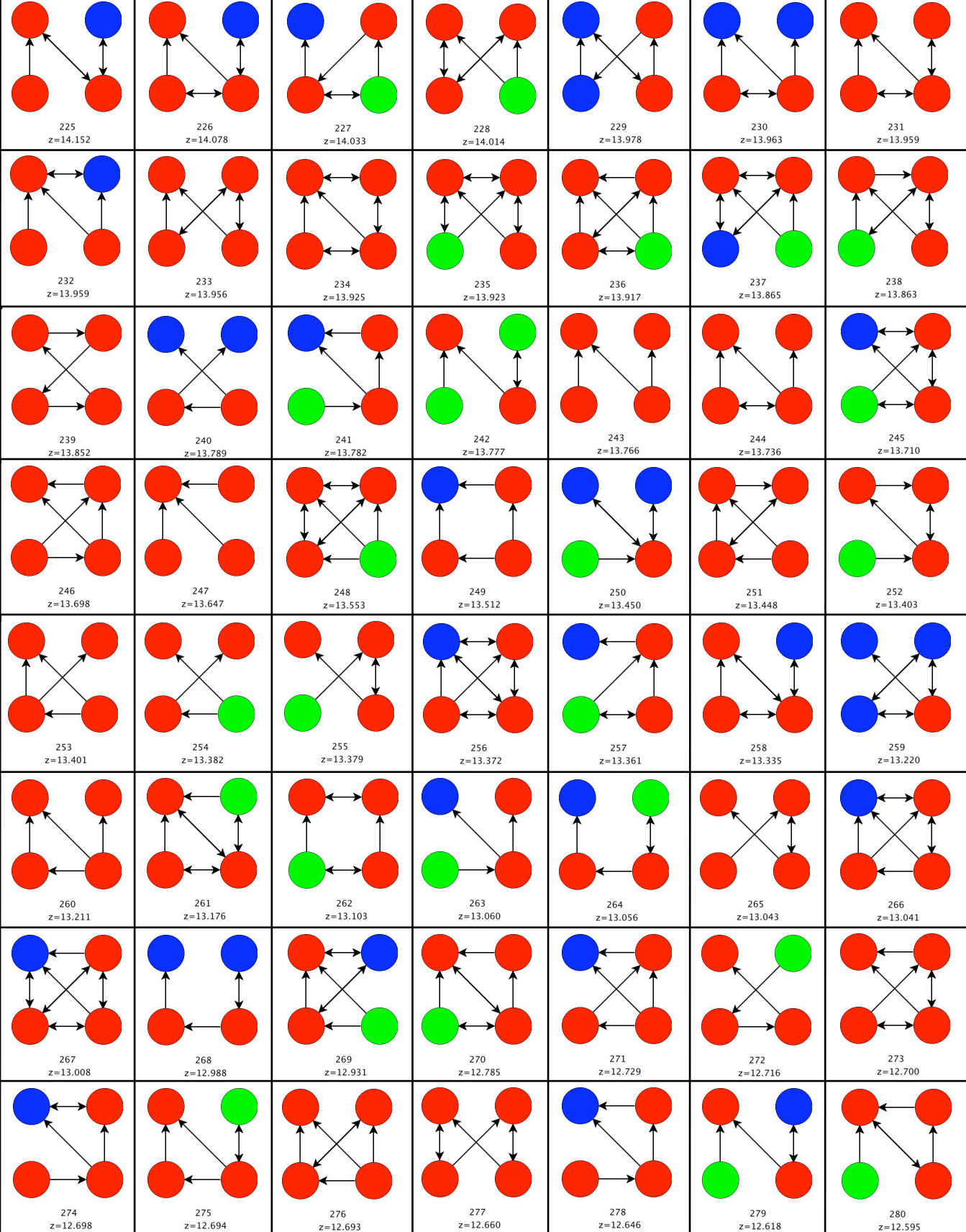

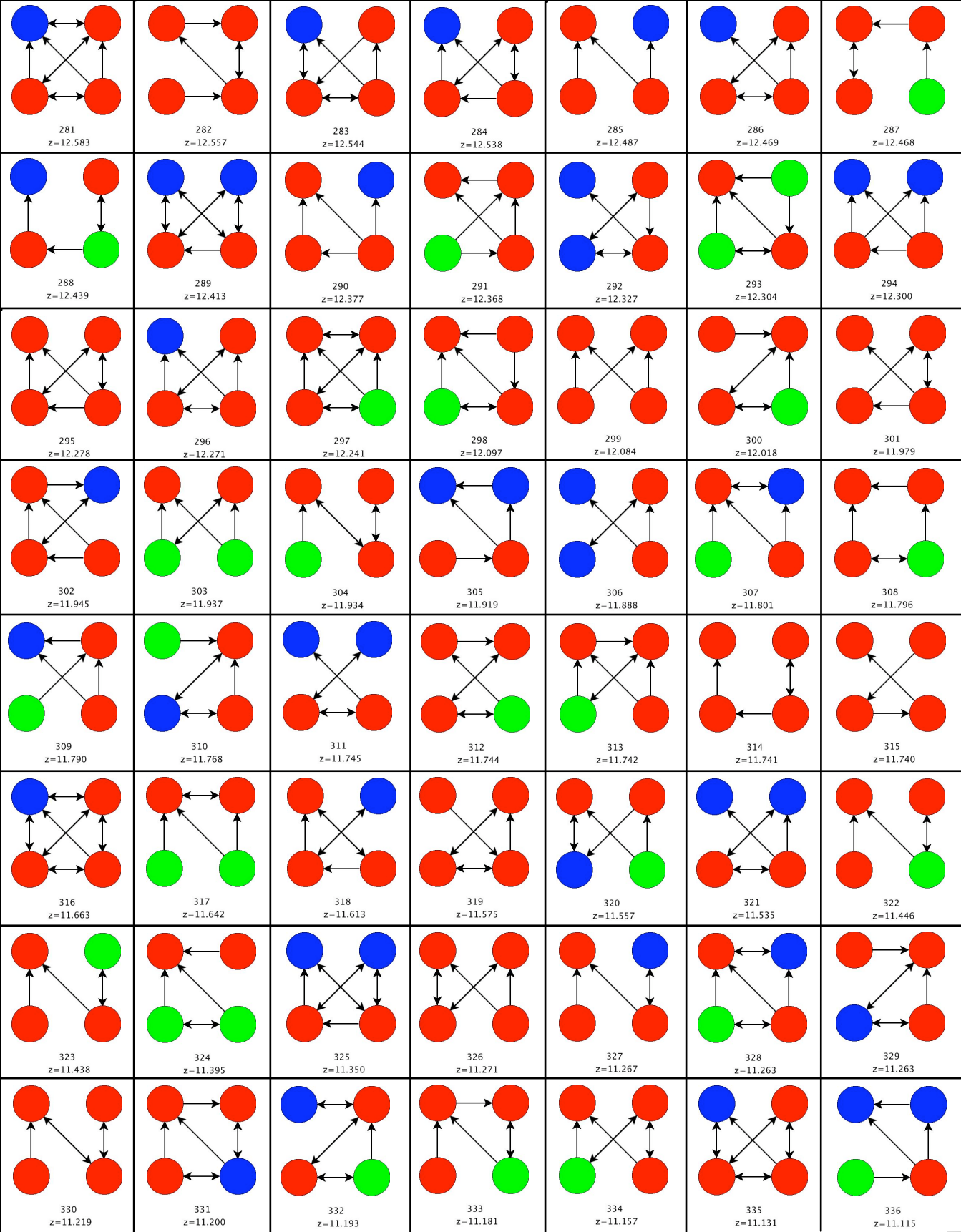

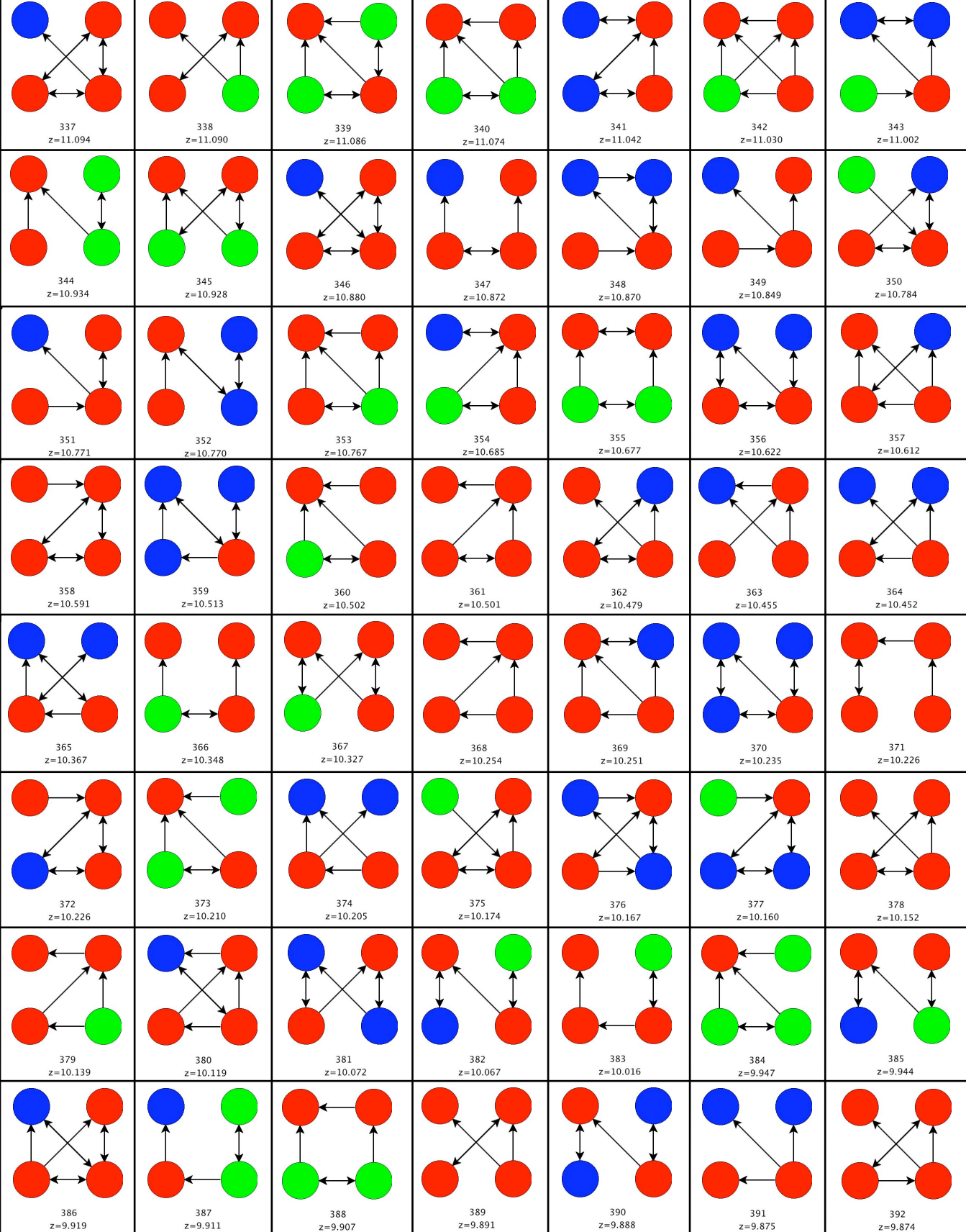

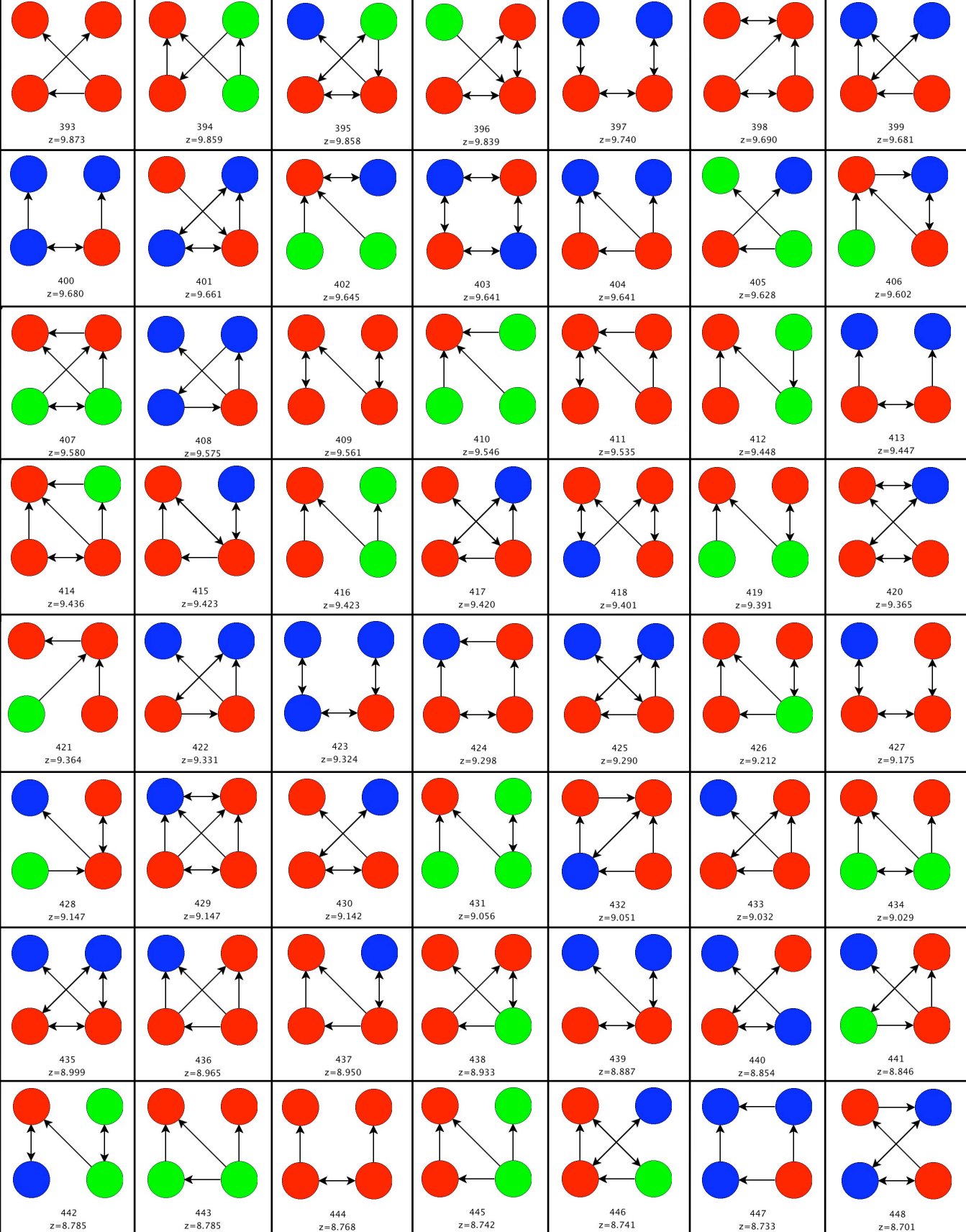

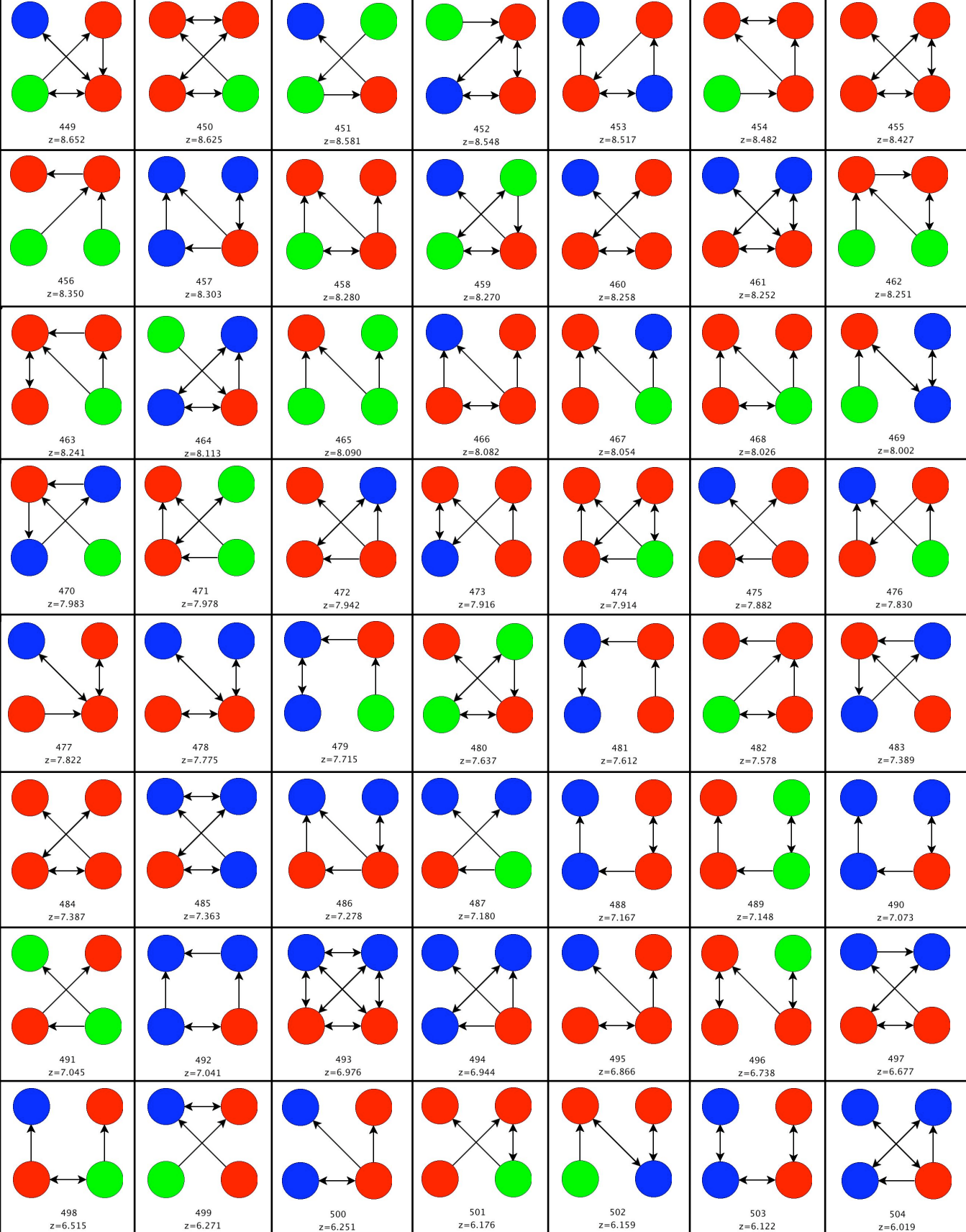

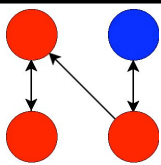

505  
z=5.740

[illegible]

Supplement: Figure S2 — Rank and -scores (un-normalized) for the 505 motifs of size 4 with single-step min P adjusted P-value P = 0.0556 for 100,000 randomizations. Each of these motifs has a (see Methods), which implies each of these motifs was more abundant in the C. elegans network than in any of the 100,000 randomized networks. But because there are 5,560 entries in that vanish, the adjusted P-value cannot be smaller than 0.0556. Increasing the number of randomizations leads to a smaller fraction of zeros in , and thus decreases the adjusted P-value of those motifs that have . (PDF) [file pone.0017013.s002.pdf]
